# G-HIV: An Integrated Long-Read Sequencing and Automated Bioinformatics Platform for Rapid and Precise HIV-1 Surveillance

**DOI:** 10.3390/microorganisms14091881

**Published:** 2026-08-24

**Authors:** Ping Fu, Zizhen Tang, Wenjie Chai, Ling Ke, Bingting Wu, Zhan Gao, Yang Huang, Dan Yuan, Qiulei Zhong, Yan Yu, Zhenxin Fan, Miao He

**Affiliations:** 1 Institute of Blood Transfusion, Chinese Academy of Medical Sciences & Peking Union Medical College, Chengdu 610052, China; fuping@ibt.pumc.edu.cn (P.F.);; 2 Southwest Bio-Resources R&D Key Laboratory of Sichuan Province, Sichuan University, Chengdu 610064, China; 3 Geneus Technologies (Chengdu) Co., Ltd., Chengdu 610219, China; 4 Center for AIDS/STD Control and Prevention, Sichuan Center for Disease Control and Prevention, Chengdu 610041, China; 5 Key Laboratory of Bioresources and Ecoenvironment (Ministry of Education), College of Life Sciences, Sichuan University, Chengdu 610064, China

**Keywords:** HIV-1, long-read sequencing, Sanger sequencing, automated bioinformatics

## Abstract

The accurate characterization of human immunodeficiency virus (HIV) genetic diversity and drug resistance is critical for effective surveillance and treatment, yet current sequencing technologies face limitations in sensitivity and scalability for community-level implementation. We present G-HIV, an integrated platform combining long-read sequencing (G-seq500) with an automated bioinformatics pipeline. G-HIV processes raw FastQ data to generate automated reports on point mutations, drug resistance predictions, viral quasispecies diversity, and haplotype networks via a two-step analytical approach. Applied to 44 HIV-1 plasma samples (42 used in the final comparison after excluding 2 samples with low-quality Sanger chromatograms), G-HIV detected 3–48 candidate minority variants per sample that were not observed by Sanger sequencing, identifying drug-resistant quasispecies in two samples with undetectable Sanger signals, and revealed mixed infection cases (e.g., inter-subtype CRF07_BC/CRF08_BC) through phylogenetic analysis. G-HIV addresses an integration of long-read sequencing with a fully automated, one-stop bioinformatics pipeline designed for frontline laboratories without specialized bioinformatics expertise—providing a scalable solution for community-based resistance surveillance and personalized therapy optimization in resource-limited settings. This research addresses an integrated long-read sequencing and automated bioinformatics platform for rapid and precise HIV-1 surveillance. G-HIV surpasses conventional approaches like Sanger sequencing in resolution, efficiency, and accessibility for community-level surveillance. By integrating long-read sequencing, streamlining workflows and eliminating the need for specialized bioinformatics expertise, G-HIV is positioned to become a new solution, providing more effective one-stop services for HIV-1 prevention and control.

## 1. Introduction

HIV remains a critical global public health threat, with an estimated 39.0 million people living with HIV at the end of 2022 [1]. In China, the epidemic persists with rising prevalence, affecting over 1.25 million individuals [2]. While antiretroviral therapy (ART) has effectively suppressed viral replication and substantially reduced morbidity and mortality [3], its long-term use is increasingly compromised by transmitted drug resistance (TDR) and viral genetic diversity [4]. Accurate genotypic drug resistance testing is pivotal for monitoring treatment efficacy and guiding prevention strategies. Consequently, the World Health Organization (WHO) recommends that countries implement nationally representative surveys to track emerging trends in HIV drug resistance [5].

However, widespread surveillance faces significant technological barriers. Sanger sequencing, the long-standing gold standard for HIV drug resistance detection, is constrained by low throughput, high per-sample costs, and insufficient sensitivity for detecting minority viral quasispecies [6]. These limitations hinder the characterization of complex heterogeneous variants, such as insertions and deletions [7,8]. Conversely, Next-generation sequencing (NGS) offers higher throughput and lower per-sample cost, enabling the detection of low-frequency variants (2–15%) [9]. However, short-read NGS platforms require computationally intensive assembly, and their error profiles—while generally low—can complicate the detection of rare variants without error-correction strategies such as unique molecular identifiers (UMIs) [9,10]. Third-generation long-read sequencing platforms such as Pacific Biosciences (PacBio) and Oxford Nanopore Technologies (ONT), have overcome read-length limitations (>20 kb), facilitating the resolution of complex genomic structures [11]. While several HIV genotypic resistance testing protocols based on these platforms have been developed, their adoption remains restricted by high operational costs and formidable bioinformatic challenges [12,13], particularly in resource-limited settings.

To bridge this gap, we leveraged the G-seq500 (Geneus Co., Ltd., Chengdu, China), a platform employing Nanopore Sequencing by Synthesis (NSS) technology [14]. Distinct from traditional nanopore methods, this technology integrates a protein nanopore with a “sequencing by synthesis” approach. During sequencing, polymerases guide the pairing of target nucleic acid strands with specially modified, charged linkers. As these linkers are drawn into the nanopore by an electric field, they generate characteristic blocking currents—distinct electrical signatures—that are algorithmically decoded into nucleic acid sequences [15,16]. The G-seq500 generates ultra-long reads (tens of kilobases) and achieves high consensus accuracy (exceeding Q50 at 50x coverage) [14], making it uniquely suited for complex genomic analyses.

Here, we present G-HIV, an integrated precise surveillance platform combining this innovative long-read sequencing technology with an automated bioinformatics pipeline. G-HIV is designed to streamline the analytical workflow, automatically generating comprehensive reports that encompass the following: (1) point mutation calling; (2) HIV-1 drug resistance (HIVDR) prediction; (3) viral quasispecies diversity assessment; and (4) haplotype network inference for phylogenetic insights. By validating the system with HIV-1 plasma samples, we demonstrate its enhanced sensitivity in detecting mixed infections, recombinant forms, and minority drug-resistant quasispecies compared to Sanger sequencing. Ultimately, this integrated system offers a streamlined, high-resolution solution, empowering frontline prevention and control laboratories to advance HIV-1 surveillance and optimize personalized treatment strategies.

## 2. Methods

### 2.1. Sample Collection

A total of 44 HIV-1 RNA positive plasma samples were included in this study, which were stored at −20 °C in our Lab collected from blood donors from Sichuan, China. A total of 44 HIV-1 RNA positive plasma samples were included in this study. Samples were collected from blood donors in Sichuan, China, between 2023 and 2024. Inclusion criteria: (1) Confirmed HIV-1 positive by nucleic acid testing or Western blot (WB); (2) the sample volume shall be sufficient for at least one round of Sanger sequencing and one round of G HIV sequencing; (3) positive result in a nested PCR pre-assay. Exclusion criteria: (1) Insufficient sample volume; and (2) low viral load resulting in a negative nested PCR pre assay result. Sample selection was performed by convenience sampling. All samples were residual plasma derived from blood donor screening and stored at −80 °C. The blood donors were typically newly diagnosed and treatment naïve.

### 2.2. Amplification and Sanger Sequencing of HIV-1 Pol Region

The method described by Zeng et al. [17] with some modifications were used to generate amplicons for G-seq500 and Sanger sequencing. In brief, HIV-1 RNA was extracted from 200 μL of plasma using a HiPure viral DNA/RNA isolation kit (Tiangen Co., Ltd., Beijing, China) and eluted to a 30 μL suspension. All the primers of nested PCR used in this study (Table S1) and the nested PCR protocols were shown in the Supplementary Materials. Amplicons, approximately 1036 bp of the *pol* region including the entire protease (PR) gene (297 nucleotides encoding 99 amino acids) and the first 244 amino acids of the reverse transcriptase (RT) region were purified and sequenced directly with inner primers by Sanger method (Tsingke Biotechnology Co., Ltd., Beijing, China).

To monitor for contamination, negative controls were included at each stage of the workflow: (1) a negative extraction control (NEC) in which PBS was processed alongside plasma samples through RNA extraction; (2) a no-template control (NTC) for the first-round RT-PCR, in which nuclease-free water replaced RNA; and (3) a no-template control for the second-round nested PCR, in which nuclease-free water replaced the first-round product. All negative controls were processed simultaneously with clinical samples and were confirmed to show no amplification. Additionally, aerosol-resistant tips and dedicated pre-PCR and post-PCR workspaces were used throughout to minimize cross-contamination risk.

### 2.3. G-seq500 Library Preparation

Library construction and sequencing complex preparation were carried out using a Universal Sample Preparation Kit (Geneus Co., Ltd., Chengdu, China). The specific steps were as follows: Firstly, 300 ng of purified amplicons were treated with the repair reagent in the kit at 20 °C for 30 min. After repairing, the amplicons were ligated with the sequencing adapter at 20 °C for 15 min. After incubation with a digestion reagent at 37 °C for 30 min, sequencing primers were added and annealed at 25 °C for 20 min. The library was then purified using magnetic beads. The 3–5 libraries were mixed at an equal molar concentration and incubated with the sequenced protein complex at 25 °C for 10 min. The sequencing complex was obtained after purification using magnetic beads.

### 2.4. G-seq500 Sequencing Procedure

The steps for sequencing using the Universal Sequencing Reagent Kit (Geneus Co., Ltd., Chengdu, China) are as follows: Firstly, power on the G-seq500 sequencer and install the Gsq500 chip. Secondly, calibrate the chip, control the temperature, and film it using the sequencing kit. Once the film is formed, add a certain dilution of the sequencing complex to the well, and add four modified deoxyribonucleotides to the well for sequencing. After the sequencing is complete, the FastQ sequencing file can be exported. Following this, the G-seq500 chip and sequencer should be cleaned using the provided cleaning kit. Finally, remove the G-seq500 chip and turn off the machine.

Due to the nature of the ligation reactions employed to create templates, the resulting products are covalently closed circular molecules that contain two complementary sequences. It is possible to generate a consensus sequence from multiple reads of both the sense and antisense strands, all derived from a single molecule. The polymerase reads are trimmed of barcode adapters to yield subreads. An approximate draft consensus is called from the subreads to provide a starting template for polishing and a final EHA (extremely high accuracy read) is generated by iteratively polishing the template using subreads. The EHA contains sequences from numerous samples mixed together and is processed to assign each sequence read to its corresponding sample based on its unique barcode.

### 2.5. Adapter Removal and Subread Extraction

To enable rapid analysis of sequencing data, we also developed an automated bioinformatic software specialized for nanopore sequencing and integrated it into the G-HIV analysis platform. G-HIV further processes the EHA data, which may consist of several samples, to separate multiplexed libraries by identifying and trimming the barcode adapter sequences using an iterative BLAST-based demultiplexing strategy, implemented on top of NCBI BLAST+ (version 2.16.0). The detection cycle begins with a low-stringency alignment using a small word size (default: 7 nucleotides) to maximize sensitivity for partial adapter matches. Each iteration dynamically adjusts alignment specificity by incrementing the word size by 2 nucleotides. The iteration stops when the proportion of unambiguous matches reaches a specific threshold (default: 0.9). By refining BLAST hits iteratively, the most possible adapter can be determined for a given read, ensuring optimal specificity. Adapter coordinates identified through alignments are used to excise flanking adapter sequences, only keeping sequences inside the target region. Reads that cannot be classified are saved separately for manual review. This approach minimized false adapter classification while preserving target viral genomic content for high-fidelity quasispecies analysis.

### 2.6. Determination of HIV-1 Subtypes, Quasispecies and TDR

**Figure 1:**
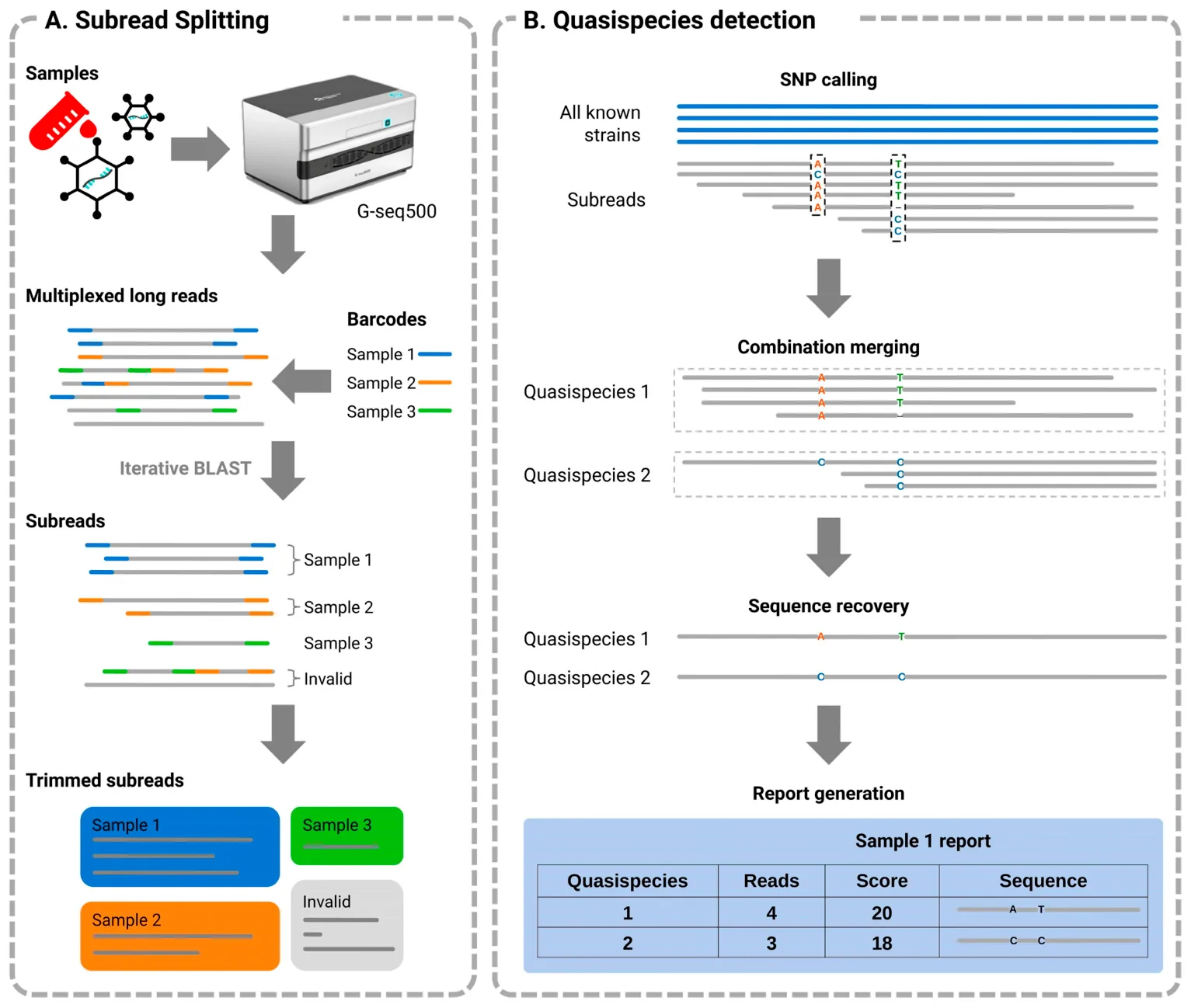
The subread splitting and quasispecies detection modules in G-HIV. (**A**) A multiplexed library is separated into individual samples and trimmed of barcode sequences through an iterative BLAST identification algorithm. (**B**) Trimmed subreads are mapped to all known reference strains and SNP sites are called. Quasispecies are inferred by merging SNP combinations and assembling the protease and partial reverse transcriptase (PR/RT) region. Colored letters represent nucleotides or gaps at polymorphic sites with International Union of Pure and Applied Chemistry codes.

Following the demarcation of subreads, G-HIV employs a two-step approach to identifying viral quasispecies within the sample (Figure 1). In the first step, sequencing reads are mapped to a reference database that encompasses all known HIV-1 groups, and the resulting alignments are processed to construct a consensus sequence. We denote the four nucleotide frequencies at position n as $F_{n,t}\left(t\in \{A,T,C,G\}\right)$ and the threshold for polymorphic sites α (default: 0.8). Due to sequencing and alignment error, most sites will have non-zero frequencies for each base even when no polymorphism exists. Therefore, the α = 0.8 threshold means that a site is only considered polymorphic when the frequency sum of true minor allele reads and noisy reads exceeds 20%. This is a deliberately conservative threshold that provides a substantial safety margin above the platform’s per-base error rate. In the k-th read, the nucleotide character mapped to position n is referred to as $R_{n,k}(R_{n,k}\in \left\{A,T,C,G,“-”\right\})$, where “−” indicates a gap. If $\underset{t\in \{A,T,C,G\}}{\max}F_{n,t}\geq \alpha$, the site at position n is considered mutation-free and ignored in subsequent analysis. Otherwise, the site is considered to be polymorphic and involved in the calling of quasispecies. The consensus sequence is simply the sequence $arg\;\underset{t}{\max}\;F_{1,t},arg\;\underset{t}{\max}\;F_{2,t},\cdots ,arg\;\underset{t}{\max}\;F_{N,t}$ for a reference sequence of length N.

In the second step, G-HIV adjusts read coordinates to ensure that all variants map to their homologous sites in reads. Potential haplotype combinations are inferred based on their co-occurrence within long reads. For the k-th read, we represent the underlying haplotype as its nucleotide characters at polymorphic sites, namely $H_{k}=\left\{\left(n,R_{n,k}\right)\underset{t\in \{A,T,C,G\}}{\max}F_{n,t}<\alpha \right\}$. Due to the relatively high error rate of nanopore sequencing, most haplotypes would contain errors. Therefore, we refine these haplotypes by merging identical combinations and collapsing low-frequency co-occurrences. After deduplication, we try to find all other haplotypes $H^{*}$ for each haplotype H, such that $\forall (n,T)\in H.\;\exists (n,T^{*})\in H^{*}.\;F_{n,T}<1-\alpha \wedge F_{n,T^{*}}\geq 1-\alpha \vee T=T^{*}$. One $H^{*}$ is selected with the largest overlap with H, and reads supporting H are marked as supporting $H^{*}$ instead. Similarly, to account for single-nucleotide alignment errors and truncated reads, every pair of reads $k_{1}$ and $k_{2}$ are compared. If $\forall \left(n,T\right)\in H_{k_{1}}.\;\exists (n,T^{*})\in H_{k_{2}}.\;T=“-”\wedge \underset{t\in \{A,T,C,G\}}{\max}F_{n_{0}-1,t}\geq \alpha \wedge \underset{t\in \{A,T,C,G\}}{\max}F_{n_{0}+1,t}\geq \alpha \vee T=T^{*}$, the combination $H_{k_{1}}$ is discarded and replaced with $H_{k_{2}}$. This allows most remaining errors in EHA reads to be corrected at the cost of inflating major allele frequency, up to the per-base error rate. This allows G-HIV to have an adaptive error profile: when the error rate is high, more polymorphic sites will be considered but later removed, enabling robust variant discovery. During error correction, G-HIV can uncover variants with a frequency lower than (1−α), if no other assignment is possible for relevant reads. If strict filtering is needed, the newly discovered variants can be removed again to ensure a consistent selection criterion. Finally, all inferred haplotype combinations are scored based on their supporting reads, ranked accordingly, and written to an output file.

Drug resistance evaluation is conducted using the Stanford HIVdb algorithm, implemented through sierra-local. Following subtyping and evaluation, samples can undergo haplotype network analysis using G-HIV. The software seamlessly integrates fastHaN for efficient network construction employing the TCS, MJN, and MSN algorithms, and extends TCSBU for high-resolution visualizations. Further enhancing its capabilities, NetST incorporates MAFFT for rapid alignment of large datasets and utilizes a DnaSP-inspired approach for robust haplotype sequence calculation.

By unifying these modules within a user-friendly environment, G-HIV streamlines the entire workflow with exceptional precise and flexibility. Consequently, it enables researchers to perform advanced analyses with unprecedented ease, making G-HIV a new tool in viral quasispecies research.

### 2.7. Phylogenetic Analyses

Reference sequences and their respective codon alignments against HXB2 were retrieved from the Los Alamos HIV Sequence Database “www.hiv.lanl.gov (accessed on 20 May 2025)”, selected based on major HIV-1 subtypes and circulating recombinant forms (CRFs) prevalent in China. A 927 bp locus of the PR/RT region, starting from HXB2 nucleotide number 2337, was extracted using the codon alignments and used in downstream analyses. Phylogenetic trees were built using IQ-TREE (version 2.3) with GTR + F + I + G4 model selected by ModelFinder. Branch support was assessed using ultrafast bootstrap with 1000 replicates. Haplotype networks are built and visualized using the integrated haplotype viewer in G-HIV. We chose the Templeton, Crandall and Sing (TCS) algorithm to build the haplotype network. The Standardized definitions of the terminology used for mixed infections were listed in the Supplementary Materials.

### 2.8. Ethics Statement

This study is compliant with the “Guidance of the Ministry of Science and Technology (MOST) of China for the Review and Approval of Human Genetic Resources” and is approved by the ethics committees of the Institute of Blood Transfusion, Chinese Academy of Medical Sciences (No. 2024016).

## 3. Results

### 3.1. Sequencing Results of G-seq500

**Figure 2:**
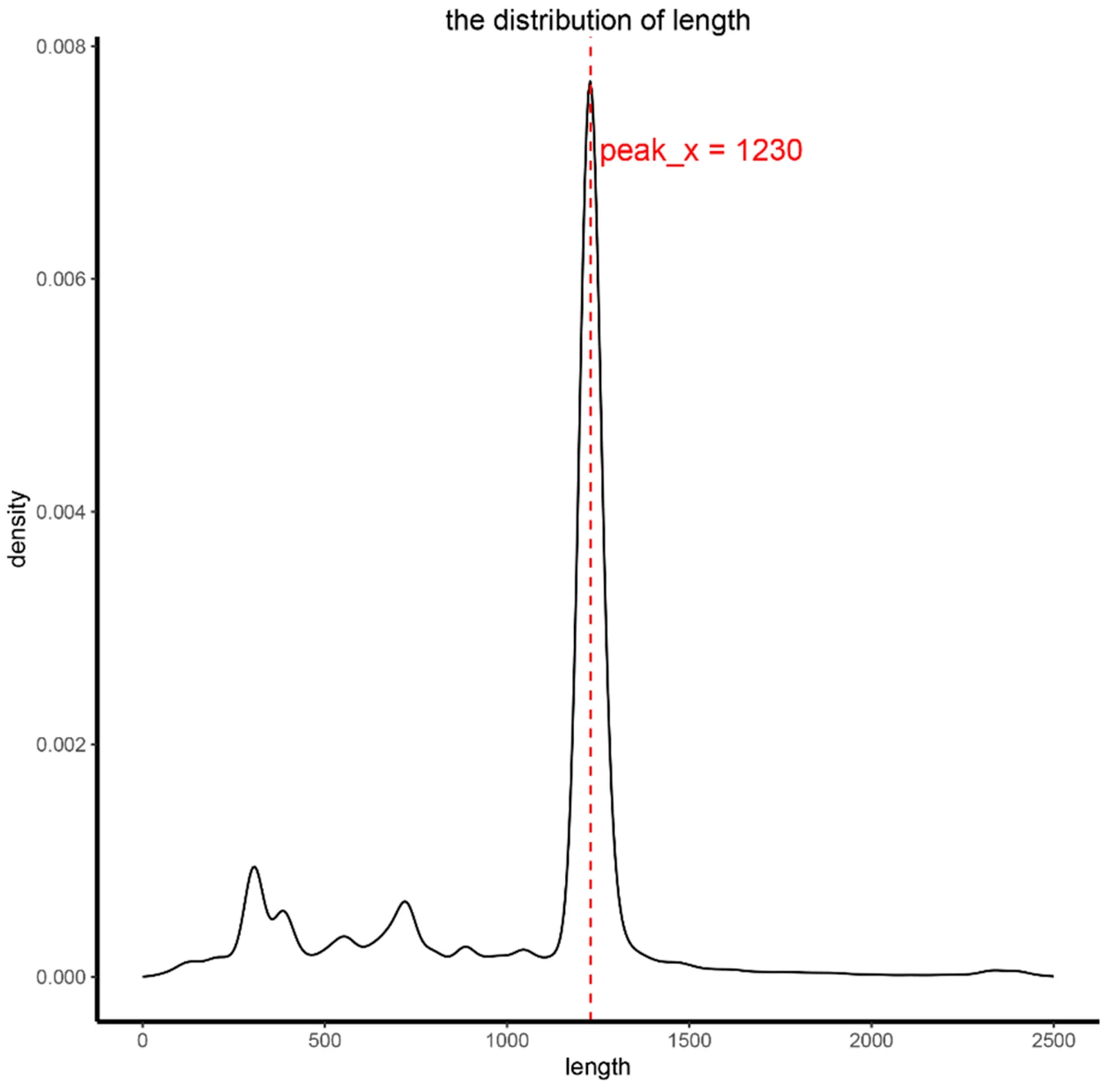
EHA sequence length distribution. The read_length_N50 is 7268 and the peak distribution of length is 1230 bp.

The biochemical system of G-seq500 consists of a nanopore protein, polymerase, the target nucleic acid strand and four types of free nucleotides with specific markers [14]. Under the action of the polymerase, the bases of the target nucleic acid strand pair sequentially with the corresponding free nucleotides. During the synthesis process, the four charged markers are drawn into the nanopore protein by the electric field, where they temporarily reside and generate characteristic blocking currents that correspond one-to-one to individual bases. By collecting the information from the current changes and using computer software and artificial intelligence algorithms to analyze the types of bases, sequencing of the target nucleic acid strand is achieved. Using a G-seq500 flow cell, a total of 7,911,724,768 bases with 1,625,296 reads were produced for 44 samples. The read_length_N50 is 7268 and the peak distribution of length is 1230 bp (Figure 2).

### 3.2. Distribution of HIV-1 Subtypes

Sequence-based analysis with Stanford HIVdb determined that the majority of quasispecies in the samples were CRFs. Two samples were discarded in the comparison because their Sanger chromatograms had low quality. Of the remaining 42 samples, the predominant HIV-1 genotype was CRF07_BC (50.0%, 21/42), followed by CRF01_AE (33.3%, 14/42) and CRF08_BC (9.5%, 4/42). Additionally, one CRF85_BC, one non-recombinant subtype B and one non-recombinant subtype C were also identified (Table S2 showed in Supplementary Materials).

### 3.3. Data Analysis by G-HIV

We analyzed the long-read sequencing data from the 42 HIV-1 RNA positive plasma samples with G-HIV, of which the lowest viral load was 8.82 × 10^2^ IU/mL. On average, 94% of total reads can be successfully demultiplexed and split into subreads. Long read lengths allowed G-HIV to recover the whole region (PR/RT), assisting in the recovery of quasispecies. Sanger sequencing results are used to cross-verify the consensus/major variant sequences from G HIV. Among the 42 samples, 6 displayed mixed peaks in their Sanger chromatograms (samples 1, 4, 11, 17, 78, and 80), leaving 36 samples without mixed peaks. All remaining 36 samples have mutations detected by G-HIV (ranging from 3 to 48) (Table 1). Details of mutations in each sample are provided in the Supplementary Materials (Table S3).

### 3.4. Mixed Infection and Quasispecies Identification by G-HIV

**Figure 3:**
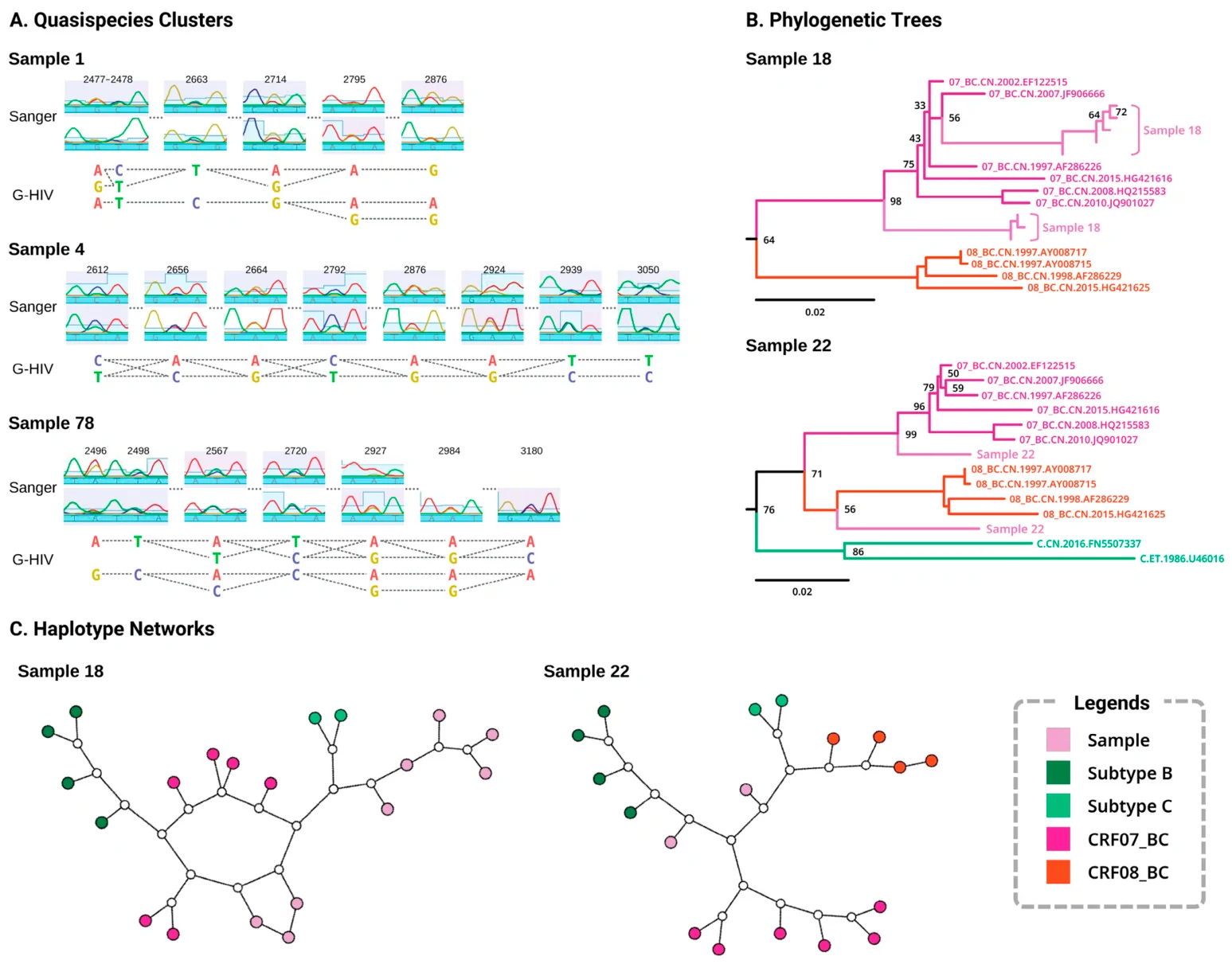
Mixed infection and quasispecies identification by G-HIV. (**A**) Sanger chromatograms and G-HIV phasing results for mutation sites in samples 1, 4 and 78. (**B**) The phylogenetic trees including 8 quasispecies in sample 18 and 2 quasispecies in sample 22. Node labels are branch support values computed with ultrafast bootstrap. Nodes without labels have 100% branch support. (**C**) Haplotype networks corresponding to the phylogenetic trees. Each colored node in the networks represents a known genotype with the color indicating its clade. Uncolored nodes indicate hypothetical genotypes that connect known sequences into a network. Lines connecting nodes represent single nucleotide differences. Node sizes are uniform and do not encode frequency information.

The recovery of the whole amplicon (PR/RT) facilitates accurate analysis of population structure, thereby shedding light on the source of new candidate minority variants (Figure 3). To illustrate this, we compared the G-HIV sequence reconstructions of three samples that were heavily heterogeneous to their Sanger chromatograms, which indicates an abundance of candidate minority variants. In sample 1 (Figure 3A), the five representative sequences extracted by G-HIV can be classified into two clusters, distinguished by site 2663. Mutation 2663T is only linked to 2795A and 2876G, which never co-occur with 2663C. In sample 4 (Figure 3A), many combinations of variable sites exist and link tightly, indicating a flat fitness landscape and a tendency for the viral population to maintain heterogeneity. Sample 78 (Figure 3A) also harbors two sub-populations. The first cluster (2496A) is also tightly connected internally. The second cluster (2496G) appears to have three unique mutations, implying a sharp fitness landscape where specific combinations at sites 2496, 2498 and 2567 might confer an advantage.

Additionally, G-HIV also detected many low-frequency genotypes which were either barely visible or undetected in Sanger chromatograms (Figure 3B). Two samples especially (18 and 22) contain genotypes from different lineages, suggesting possible mix infection. We further investigated the three possible cases of superinfection using the integrated haplotype network inference functionality. The phylogenetic trees and haplotype networks are displayed in Figure 3C. In sample 18, the genotypes form two clades in the CRF07_BC clade. In sample 22, one genotype locates in the CRF07_BC clade and the other locates in the CRF08_BC clade. Similarly, in the haplotype network, the nodes representing those genotypes are not clustered together, instead forming two clusters in different parts of the network, highlighting the existence of intra-subtype mixed infection.

### 3.5. Drug Resistance by G-HIV

Despite the high diversity of virus-candidate minority variants, it is notable that most samples do not confer significant drug resistance—only four samples contain known mutations related to intermediate or high-level resistance (Table 2). All other samples confer no or low-level resistance. Three of the four drug-resistant samples harbor quasispecies with varying degrees of drug resistance and drug-susceptible quasispecies, providing insight into molecular evolution under antiviral treatment.

## 4. Discussion

Transmitted drug resistance (TDR) poses a growing challenge to HIV epidemic control in China. The prevalence of TDR among ART-naïve individuals has risen from moderate levels (7.8%) in 2020–2022 to 11.4% in 2023 [4,18]. On top of this alarming trend, frontline HIV prevention and control are constrained by infrastructural limitations and a lack of specialized operational capacity. Consequently, resistance monitoring is often centralized in provincial or national laboratories, leading to significant turnaround times that impede the timely optimization of antiretroviral therapy (ART) regimens.

Existing sequencing technologies struggle to meet these grassroots needs. While Sanger sequencing serves as the conventional standard, it fails to derive linkage information from chromatograms, making it difficult to distinguish co-infections or superinfections from simple mixed populations. Its limited sensitivity also hinders the detection of low-frequency variants, preventing the construction of accurate molecular transmission networks in complex infection scenarios [19,20]. Conversely, while next-generation sequencing (NGS) offers higher sensitivity, the complexity of short-read assembly and the requirement for bioinformatic expertise create high barriers to entry for frontline prevention and control laboratories [21,22].

To address these challenges, we developed G-HIV, a streamlined platform designed to make advanced sequencing accessible to frontline healthcare workers. Unlike traditional workflows that require extensive bioinformatic skills, G-HIV integrates long-read sequencing (G-seq500) with an automated analysis pipeline. This “sample-to-report” solution enables the generation of comprehensive results—including point mutations, drug resistance profiles, quasispecies diversity, and haplotype networks—within one day. This represents a significant efficiency gain over centralized testing models, which can take up to two months. By automating complex data processing, G-HIV effectively bridges the gap between sophisticated genomic technology and highly efficient and precise frontline surveillance needs.

A key advantage of G-HIV is its enhanced sensitivity in detecting viral heterogeneity. In our study, G-HIV identified candidate minority variants (ranging from 3 to 48 per sample) in 39 samples that were not observed by Sanger sequencing. Even in the low viral load sample (such as sample 79, 8.82 × 10^2^ IU/mL), G-HIV could detect 10 candidate minority variants that were not observed by Sanger sequencing. This capability is clinically critical, as minority drug-resistant quasispecies—undetectable at baseline by Sanger methods—can rapidly dominate the viral population under selective pressure from ART, leading to early treatment failure [13,23]. For instance, in samples 69 and 76, G-HIV detected drug-resistant candidate minority variants that were masked in Sanger chromatograms, highlighting its potential to guide more effective initial treatment selection.

In the absence of longitudinal clinical data, the temporal sequence of infection (simultaneous vs. sequential) cannot be determined from cross-sectional sequencing data alone. We therefore use the term ‘mixed infection’ to describe samples containing genetically distinct viral populations, and we note that these cases could represent either co-infection or superinfection. G-HIV provides unprecedented resolution for distinguishing co-infections and superinfections. Traditional bulk sequencing often obscures the dynamic nature of viral populations. By leveraging long reads to recover full-length haplotypes, G-HIV allows for the direct visualization of population structures. Our phylogenetic and haplotype network analyses revealed intra-subtype co-infections in samples 18 and 22, scenarios that appeared as ambiguous “mixed peaks” or were entirely not obverted by Sanger sequencing. Accurately distinguishing between co-infection (simultaneous infection) and superinfection (sequential infection) is vital for molecular epidemiology. It provides the high-resolution evidence needed to trace transmission chains accurately, which is increasingly important for forensic epidemiology and clarifying transmission relationships in legal contexts [24].

G-HIV may not be the most sensitive sequencing platform available; rather, it is the integration of long-read sequencing with a fully automated, one-stop bioinformatics pipeline designed for frontline laboratories without specialized bioinformatics expertise. The G-seq500’s long reads enable direct recovery of full-length haplotypes without short-read assembly, and the automated pipeline eliminates the need for expert intervention—a combination not offered by other workflows. As provinces in China strive to build precise molecular monitoring networks, the ability to identify key transmission clusters and high-risk individuals is paramount. G-HIV supports this by offering a scalable, cost-effective solution for detecting recombinant forms and monitoring the evolution of multi-drug-resistant strains. Early detection of these events allows for the rapid adjustment of preventative measures and treatment plans, thereby curbing the spread of resistance.

The limitations of this study should also be acknowledged. First, while G-HIV demonstrates high agreement with Sanger sequencing for dominant candidate minority variants, the sample size in this validation study was relatively small. The validation cohort, while encompassing six distinct HIV-1 genotypes, was limited to 42 samples from a single province in southwestern China. Primer performance, amplification efficiency, and long-read alignment accuracy should be evaluated across a broader spectrum of geographically and genetically diverse HIV-1 strains to exclude subtype-dependent amplification bias or primer dropout. Future multi-center studies with larger cohorts spanning diverse geographic regions and clinical settings—including treatment-experienced patients, vertically infected children, and specimens from other epidemic regions—are planned to further establish the platform’s robustness and generalizability. Second, although the cost per sample is lower than traditional NGS, it may still present a burden for extremely resource-limited settings, though this is partially offset by the reduced need for specialized personnel and equipment. Third, the enhanced sensitivity is limited to Sanger sequencing. We acknowledge that a direct benchmarking comparison with established NGS platforms such as Illumina MiSeq (ideally with unique molecular identifiers) would provide additional validation of G-HIV’s analytical performance. Such a head-to-head comparison is planned for future studies. We also acknowledge that the combination of nested PCR amplification and long-read sequencing introduces potential sources of error, including PCR recombination, amplification bias, and residual sequencing errors. Although the G-seq500’s Circular Consensus Sequencing (CCS) approach substantially reduces per-read error rates through intra-molecular consensus (achieving Q50 + accuracy), it does not employ unique molecular identifiers (UMIs) to correct for errors introduced during PCR amplification. Without UMIs or orthogonal validation methods such as ddPCR, the candidate minority variants identified exclusively by G-HIV should be interpreted with appropriate caution. Future studies incorporating UMI-based approaches and orthogonal validation of key variants are planned to further strengthen confidence in these calls. Fourth, in this validation study, technical replicates (independent RNA extraction or repeated RT-PCR) were not systematically performed for all samples due to the insufficient sample volume. Future validation studies will incorporate replicate testing to assess the reproducibility of candidate minority variant calls. Fifth, conventional testing includes sequencing of the first 1.5 kb of the *pol* gene to capture the protease and reverse transcriptase coding regions for the prediction of PI, NRTI, and NNRTI resistance [25]. However, in 2019, the WHO recommended the 2nd generation integrase strand transfer inhibitors (INSTIs)—dolutegravir (DTG)—as the third agent of the first-line regimen [26]. For patients who require INSTI resistance testing, the integrase coding region of the *pol* gene should be sequenced as well [25]. Additionally, the HIV-1 envelope glycoprotein (*env*; encoding gp120/gp41) is the target of entry and fusion inhibitors, including Enfuvirtide, Maraviroc, Fostemsavir, and Ibalizumab, as well as emerging broadly neutralizing antibodies (bNAbs) [26,27]. Exclusion of *env* therefore precludes the assessment of resistance to these important therapeutic modalities. Restricting *pol* coverage to the protease and reverse transcriptase regions omits the integrase (*IN*) gene, creating a blind spot for resistance surveillance of current frontline therapies.

In conclusion, G-HIV surpasses conventional Sanger sequencing in resolution, efficiency, and accessibility. By providing a user-friendly, one-stop solution for precise sequencing, it holds the potential to decentralize HIV-1 resistance monitoring. This shift towards frontline surveillance is essential for the timely and effective control of HIV-1 transmission and drug resistance in China and other high-burden regions.

## Figures and Tables

**Table 1:** List of subtypes and number of mutations in each sample.

Sample No	Viral Load (IU/mL)	Subtype		Mutation Sites		
		Sanger	G-HIV	Sanger	G-HIV	Present in G-HIV, Not Detected by Sanger
1	8.85 × 10^3^	CRF08_BC	CRF08_BC	6	24	18
4	3.64 × 10^4^	CRF07_BC	CRF07_BC	8	24	16
5	2.03 × 10^4^	B	B	0	19	19
6	2.24 × 10^5^	CRF07_BC	CRF07_BC	0	3	3
8	Unknown	CRF07_BC	CRF07_BC	0	6	6
9	Unknown	CRF07_BC	CRF07_BC	0	5	5
10	Unknown	CRF08_BC	CRF08_BC	0	11	11
11	Unknown	CRF07_BC	CRF07_BC	1	13	12
12	Unknown	CRF01_AE	CRF01_AE	0	15	15
13	Unknown	CRF01_AE	CRF01_AE	0	8	8
14	2.60 × 10^3^	C	C	0	15	15
15	Unknown	CRF01_AE	CRF01_AE	0	3	3
17	Unknown	CRF07_BC	CRF07_BC	1	10	9
18	1.17 × 10^4^	CRF07_BC	CRF07_BC	0	42	42
19	Unknown	CRF08_BC	CRF08_BC	0	10	10
22	2.04 × 10^5^	CRF07_BC	CRF07_BC CRF08_BC	0	48	48
23	5.71 × 10^3^	CRF01_AE	CRF01_AE	0	8	8
25	7.60 × 10^5^	CRF01_AE	CRF01_AE	0	8	8
26	>Titer max	CRF01_AE	CRF01_AE	0	7	7
27	1.38 × 10^3^	CRF85_BC	CRF85_BC	0	12	12
28	2.67 × 10^5^	CRF01_AE	CRF01_AE	0	12	12
38	2.02 × 10^3^	CRF01_AE	CRF01_AE	0	7	7
41	5.60 × 10^5^	CRF07_BC	CRF07_BC	0	11	11
42	1.38 × 10^4^	CRF07_BC	CRF07_BC	0	10	10
43	4.92 × 10^5^	CRF07_BC	CRF07_BC	0	11	11
50	2.72 × 10^5^	CRF07_BC	CRF07_BC	0	11	11
51	2.97 × 10^5^	CRF01_AE	CRF01_AE	0	7	7
55	5.14 × 10^4^	CRF07_BC	CRF07_BC	0	7	7
56	5.12 × 10^5^	CRF07_BC	CRF07_BC	0	4	4
59	1.41 × 10^6^	CRF01_AE	CRF01_AE	0	8	8
61	5.00 × 10^4^	CRF07_BC	CRF07_BC	0	7	7
62	2.03 × 10^4^	CRF07_BC	CRF07_BC	0	4	4
69	1.35 × 10^6^	CRF01_AE	CRF01_AE	0	3	3
71	1.74 × 10^5^	CRF01_AE	CRF01_AE	0	14	14
72	5.21 × 10^4^	CRF01_AE	CRF01_AE	0	9	9
73	4.52 × 10^3^	CRF07_BC	CRF07_BC	0	13	13
75	7.89 × 10^4^	CRF08_BC	CRF08_BC	0	11	11
76	7.23 × 10^4^	CRF07_BC	CRF07_BC	0	6	6
78	2.77 × 10^3^	CRF07_BC	CRF07_BC	7	15	8
79	8.82 × 10^2^	CRF01_AE	CRF01_AE	0	10	10
80	1.63 × 10^5^	CRF07_BC	CRF07_BC	2	15	13
82	4.16 × 10^4^	CRF07_BC	CRF07_BC	0	9	9

**Table 2:** List of the four samples with drug resistance.

Sample No.	Number of Quasispecies	High Drug Resistance	Moderate Drug Resistance	Detectable by Sanger Sequencing
69	2	EFV, NVP	–	Yes
		EFV, NVP	–	No
71	3	NVP	EFV, ETR, RPV	Yes
		–	–	No
		–	–	No
73	3	EFV, NVP	–	Yes
		EFV, NVP	–	Yes
		–	–	No
76	3	AZT, EFV, NVP	D4T, DOR	Yes
		AZT, EFV, NVP	D4T, DOR	No
		AZT	D4T, EFV, NVP	No

Note: EFV: Efavirenz; NVP: Nevirapine; ETR: Etravirine; RPV: Rilpivirine; AZT: Zidovudine; DOR: Doravirine; D4T: Stavudine.

## Data Availability

All the code utilized for development and validation is available at https://gitee.com/sculab/G-HIV (accessed on 18 August 2026) to anyone who wishes to utilize it for any purpose following publication. All raw imaging data will be made available upon request by contacting hemiao@ibt.pumc.edu.cn immediately after publication.

## Supplementary Materials

The following supporting information can be downloaded at: https://www.mdpi.com/article/10.3390/microorganisms14091881/s1, Table S1: Primers of nested PCR for 1036 bp amplicons. Table S2: The distribution of HIV-1 genotypes by phylogenetic tree. Table S3: Details of mutations in each sample.
