# Supplementary material for: G-HIV: An Integrated Long-Read Sequencing and Automated Bioinformatics Platform for Rapid and Precise HIV-1 Surveillance
Source: Microorganisms. 2026 Aug 24;14(9):1881. doi: 10.3390/microorganisms14091881 (PMC13609792; doi:10.3390/microorganisms14091881)
Supplement: Supplementary file 1 [file microorganisms-14-01881-s001.zip › Supplementary Tables S1 and S2.pdf]

Supplementary Materials

1. Table S1

Table S1. Primers of nested PCR for 1036 bp amplicons.

	Primer name	Nucleotide Position in HXB2 (bp)	5′-3′ Sequence
Outer	MAW 26	2029-2050	5′-TGGAAATGTGGAAAGGAAGGAC-3′
Primers	RT21	3509-3539	5′-CTGTATTTCTGCTATTAAGTCTTTTGATGGG-3′
Inner	Pro-1	2147-2166	5′-CAGAGCCAACAGCCCCACCA-3′
Primers	RT27	3299-3327	5′-CTTCTGTATATCATTGACAGTCCAGCT-3′

2. Nested PCR protocol:

For the primary RT-PCR, 10 μL of purified HIV-1 RNA from each sample were firstly incubated for 5 min at 65 °C to unfold the RNA, followed by stabilization of RNA by incubation on ice.

Following the incubation, 5 μL of unfold HIV-1 RNA, 10 nmol of each outer primer (MAW 26 and RT21), 2× FastKing One Step RT-PCR MasterMix and 25×RT-PCR Enzyme Mix (Tiangen, Inc., Beijing, China) were added, as well as nuclease free water for a final volume of 25 μL. The first round PCR amplifications were run under the following conditions in a Thermal Cycler (Biometra): one cycle of 42 ° C for 30 min, 94°C for 3 minutes and then 35 cycles of 94°C for 30 seconds, 55°C for 30 seconds, and 72°C for 1 minute 30 seconds followed by 72°C for 5 minutes.

The second round PCR taking 1 μL of the first round PCR product as template was amplified with 25 nmol of each inner primers (Pro-1 and RT 27), 2× UltraHiFi Mix II (Tiangen, Inc., Beijing, China), as well as nuclease free water for a final volume of 50 μL, under the following conditions: one cycle of 98°C for 30 second, and then 35 cycles of 98°C for 10 seconds, 63°C for 20 seconds, and 72°C 1 minute, followed by 72°C 5 minutes.

3. Definitions and classification criteria of mixed infections

(1). Classification criteria.

- Mixed infection (umbrella term): The presence of more than one genetically distinct HIV-1 strain within a single host, as detected by sequencing. This is the preferred descriptive term when clinical timing information is unavailable.
- Co-infection: Simultaneous or near-simultaneous infection by two or more genetically distinct HIV-1 strains, typically occurring around the time of primary infection. This can be confirmed only when clinical and epidemiological evidence indicates that the infections were acquired concurrently.
- Superinfection: Sequential infection by a second, genetically distinct HIV-1 strain in an individual who is already infected. This requires evidence of pre-existing infection followed by acquisition of a new strain, typically supported by longitudinal clinical data.
- Intra-subtype mixed infection: Mixed infection involving two or more strains of the same subtype or CRF (e.g., two distinct CRF07_BC strains).
- Inter-subtype mixed infection: Mixed infection involving strains from different subtypes or CRFs (e.g., CRF07_BC and CRF08_BC).

(2). Classification criteria.

Samples were classified as mixed infections based on the following criteria: (1) Recovery of two or more distinct haplotypes from the same sample, where 'distinct' is defined as haplotypes differing at ≥2 polymorphic sites; (2) Phylogenetic placement of these haplotypes in separate clades or lineages, as determined by IQ-TREE analysis with ultrafast bootstrap support ≥95%; (3) Topological separation in the TCS haplotype network, where distinct haplotypes form unconnected or distantly connected clusters; and (4) Corroborating evidence of mixed populations in Sanger chromatograms (mixed peaks at polymorphic positions).

Samples meeting criteria (1) - (3) were classified as mixed infections based on sequencing evidence alone. The sub-classification into co-infection versus superinfection requires longitudinal clinical data on the timing of infection, which was not available for this study.

Therefore, we use the term 'mixed infection' as the default classification and discuss co-infection and superinfection as biological possibilities.

4. Table S2

Table S2. The distribution of HIV-1 genotypes by phylogenetic tree

Subtype/CRFs	Number	Percentage (%)
CRF07_BC	21	50
CRF01_AE	14	33.3
CRF08_BC	4	9.5
CRF85_BC	1	2.4
B	1	2.4
C	1	2.4
